# Social network size and cognitive resilience to neurodegeneration in the Framingham Heart Study

**DOI:** 10.1002/alz70860_097211

**Published:** 2025-12-23

**Authors:** Kristi Ho, Rhoda Au, Phillip H Hwang

**Affiliations:** ^1^ Boston University Chobanian & Avedisian School of Medicine, Boston, MA, USA; ^2^ Boston University School of Public Health, Boston, MA, USA; ^3^ Framingham Heart Study, Framingham, MA, USA; ^4^ The Framingham Heart Study, Framingham, MA, USA

## Abstract

**Background:**

Social stimulation from having large networks may contribute to cognitive resilience; however, limited research has assessed whether this relationship differs across the life course. We examined the relationship between social network size and resilience to neurodegeneration in memory, language, and executive function among midlife and late‐life adults.

**Method:**

The sample included 1,602 participants from the Framingham Heart Study Offspring cohort who completed the Berkman‐Syme Social Network Index (SNI) questionnaire, had available plasma total tau (t‐tau) measures at Exam 8, and had calculated neuropsychological (NP) factor scores in memory, language, and executive function. Social network size was operationalized through a calculated SNI score and categorized into small, medium, and large networks. Cognitive resilience was operationalized using a residual approach by regressing each NP factor score on the plasma t‐tau measure. Linear regression was used to assess associations between social network size and cognitive resilience, including stratifying the results by midlife (47‐64 years) and late‐life (65‐91 years) groups, and adjusting for age, sex, education, APOE ε4 allele carrier status, time between the most recent NP exam visit date and Exam 8 date, physical activity, smoking status, and depressive symptoms.

**Result:**

The sample average age was 67±8.3 years with most participants identifying as female (*n* = 880; 55%). By age group, more were in the late‐life group (*n* = 918; 57%) compared to the midlife group (*n* = 684; 43%). Large social network size was significantly associated with greater resilience to neurodegeneration in memory compared to small social networks in the fully adjusted model (β = 0.070; 95% CI = 0.007 – 0.133). There were no significant associations between large social networks and resilience to neurodegeneration in language or executive function, but large social networks were associated with resilience to neurodegeneration in executive function among the midlife group (β = 0.110; 95% CI = 0.010 – 0.210).

**Conclusion:**

Results suggest that having large social networks are protective for memory impairment in elderly adults, and for better executive function at midlife. Additional studies in more diverse samples are needed to replicate and generalize our findings to other groups.

